# *FLT3* Amplification as Double Minute Chromosomes in a Patient with Chronic Myelomonocytic Leukemia

**DOI:** 10.1155/2021/9932837

**Published:** 2021-06-05

**Authors:** Heyang Zhang, Xiaoxue Wang, Shibo Li, Xianfu Wang, Xianglan Lu, Ming Li, Hua Wang, Ying Liu, Hui Pang, Lijun Zhang

**Affiliations:** ^1^Department of Hematology, The First Hospital of China Medical University, Shenyang, Liaoning, China; ^2^Department of Pediatrics, University of Oklahoma Health Sciences Center, Oklahoma City, Oklahoma, USA; ^3^Division of Genetics, Department of Pediatrics, Loma Linda University, Loma Linda, CA, USA

## Abstract

Double minute chromosomes (dmins) are a form of gene amplification presenting as small spherical paired chromatin bodies. Dmins are rare in hematologic malignancies and are generally associated with a poor prognosis. Some case reports identified *MYC* or *MLL* gene amplification performing as dmin in myeloid neoplasms. *FLT3* (*FMS*-related tyrosine kinase 3) acts as an oncogene in myeloid neoplasms which is associated with several signal transduction pathways. Genomic amplification of FLT3 has not been reported in hematological disease. The current study attempts to demonstrate the existence of double minute chromosomes via *FLT3* gene amplification in a patient diagnosed with chronic myelomonocytic leukemia (CMML). Routine G-banded karyotype, array-based comparative genomic hybridization, and fluorescence in situ hybridization analyses were used to characterize the cytogenetic abnormality in the patient's bone marrow. *FLT3* amplification as dmins in a patient with CMML was revealed. This case study reports a rare double minute chromosome via *FLT3* amplification in CMML by using array-based comparative genomic hybridization and fluorescence in situ hybridization analyses. The study also proposed another possible mechanism of *FLT3* genes in leukemogenesis.

## 1. Introduction

Double minute chromosomes (dmins) are cytogenetic indicators of extrachromosomal gene amplification which usually act as small spherical paired chromatin particles [1]. Dmin has been found in a lot of human neoplasia especially solid tumors [2]. The presence of dmin often correlates with a poor prognosis in tumors [3]. The occurrence of dmin is relatively low in hematologic malignancies. The frequencies of dmin in acute myeloid leukemia (AML) range from 0.3% to 2.8% [4]. The role of dmin in leukemogenesis is still not clear. It is generally considered to be involved in tumorigenesis and associated with an upregulated oncogene expression which may be linked to poor outcomes [5]. Several published literatures revealed that some oncogenes, such as MYC and MLL, have been identified to be amplified on dmins in AML and myelodysplastic syndrome (MDS) [6].

*FLT3* (FMS-related tyrosine kinase 3) located on chromosome 13q12.2 encodes a receptor tyrosine kinase (RTK) that activates the Ras and PI3 kinase pathway leading to the increased proliferation and inhibition of apoptosis in hemopoietic progenitor cells [7]. The oncogene activation of *FLT3* in hematological malignancies is mainly manifested through internal tandem duplication which may result in a poor prognosis [8]. Genomic amplification of *FLT3* has been reported in solid tumors including colorectal cancer, breast cancer, and gastric cancer [9]. However, no exhibited amplification of *FLT3* on dmins has been reported in hematological malignancies. Here, to our best knowledge, we present the first case of amplification encompassing the *FLT3* gene acting as dmin in a patient with chronic myelomonocytic leukemia (CMML). This study was approved by the Institutional Review Board (IRB) of the University of Oklahoma Health Science Center (OUHSC).

## 2. Material and Methods

### 2.1. Cytogenetics

Overnight culture of peripheral blood was prepared according to standard laboratory protocols. Karyotype analysis was performed by the G-banding technique. A total of 20 cells were analyzed. The cytogenetic abnormalities were described according to the International System for Human Cytogenetic Nomenclature (ISCN).

### 2.2. Oligonucleotide aCGH Assay

Genomic DNA was purified from the peripheral blood samples using the Maxwell RSC Blood DNA kit (Promega) as per the manufacturer's recommendations. Array comparative genomic hybridization (CGH) was performed following the standard protocol provided by Agilent Technologies (Agilent Technologies, Santa Clara, CA, United States). In brief, the patient genomic DNA and gender-matched reference genomic DNA were labeled with cyanine 5 (Cy5) and cyanine 3 (Cy3), respectively. Equal quantities of labeled DNA products were mixed together and loaded onto an Agilent 2 × 400 k CGH chip, which is built based on GRCh37/hg19 with 1 kb median probe spacing. Raw data were analyzed using CytoGenomics 5.0 software (Agilent Technologies, Santa Clara, CA, United States).

### 2.3. FISH

Subsequent FISH analyses were performed to confirm the amplification detected by array CGH. Commercially available *FLT3*/CON13 FISH probe (Empire Genomics, *FLT3*-CHR13-20-ORAQ) was applied on the cultured blood cells.

### 2.4. Case Presentation

**Figure 1:**
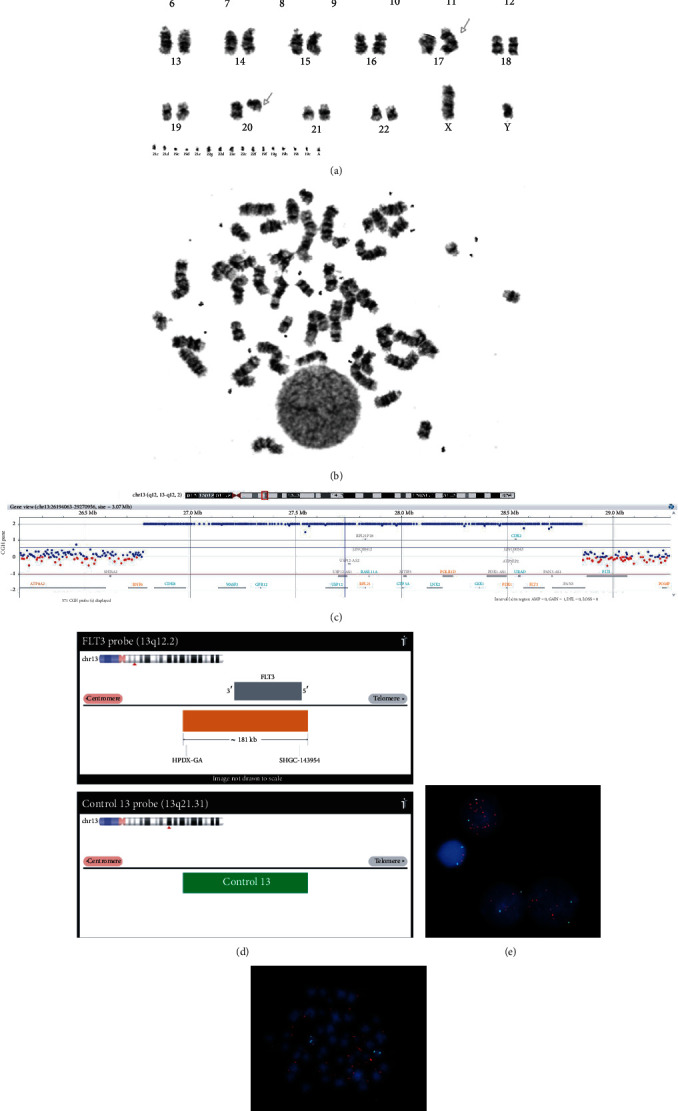
Array CGH, conventional cytogenetic, and FISH analyses with a *FLT3*/CON13 probe. Karyotype (a) and metaphase spread (b) showing 46,XY,i(17)(q10),del(20)(q11.2q13.3) 1~20dmin. (c) Array comparative genomic hybridization (CGH) was performed following the standard protocol provided by Agilent Technologies (Agilent Technologies, Santa Clara, CA, United States) showing the region of amplification. Equal quantities of labeled DNA products were mixed together and loaded onto Agilent'3 2∗400 k CGH chip, which is built based on GRCh37/hg19 with 1 kb median probe spacing. (d) *FLT3/*CON13 FISH probe. Interphase FISH (e) and metaphase FISH (f) showing numerous copies of *FLT3*.

The patient is a 64-year-old male who was admitted to the hospital because of an abnormal finding of complete blood count (CBC), with a white blood cell (WBC) count of 14.58 × 10^9^/L, a hemoglobin (HGB) level of 77 g/L, a platelet level of 12 × 10^9^/L, and 16.6% circulating blasts. The bone marrow was hypercellular, with myeloid hyperplasia, trilineage dysplasia, and 13.3% blasts. Flow cytometric immune phenotyping performed on the bone marrow aspirate revealed 24% monocytic cells and 23% myeloid blasts expressing partial CD4, CD13, CD33, CD34, CD38, CD117, and HLA-DR and moderate CD45. Based on the laboratory findings above, the patient was diagnosed with CMML-2. Routine chromosomal analysis was performed on blood. The results revealed two related abnormal cell lines. The first line, seen in 8 cells, showed an isochromosome composed of 17q and a deletion of 20q. The second line, seen in 12 cells, had the same abnormalities observed in the first line plus 1 to 20 dmins. The karyotype result was designated as 46,XY,i(17)(q10),del(20)(q11.2q13.3)[8]/46,idem,1~20dmin[12] (Figures 1(a) and 1(b)). In order to assess the origin of the dmin, whole genome array comparative genomic hybridization (aCGH) was performed. The results were described as array [hg19]13q12.13q12.2(26781583_28851524) amp, 17p13.3p11.2(47546_21182807) x1,17p11.2p11.1(21715685_22218445) x3, 17q11.1q25.3(25375449_81108062) x3,20q11.21q13.31(31798183_55438537) x1. These results confirmed i(17) and del(20q) identified by conventional cytogenetics. In particular, amplification of chromosome 13q12.13q12.2 was identified. The size of the amplified region is ~2 Mb, including 4 disease-causing genes, *RNF6*, *RPL21*, *POLRI1*, and *FLT3* likely resulting from the amplification of this region. The amplified region includes *POLR1D*, *FLT3*, *RPL21*, *PDX1*, and *CDK8* (Figure 1(c)). To determine whether the dmin identified in this case is derived from this region, fluorescence in situ hybridization (FISH) analysis using the probe specifically designed to detect *FLT3* amplifications and deletions was applied on the cultured blood cells. The *FLT3*/CON13 probe was provided by Empire Genomics. Probe design is shown in Figure 1(d). The *FLT3* gene was labeled as orange; the control 13 probe located at the 13q21.31 region was labeled with aqua fluorescence dye. A total of 200 cells were analyzed, and ~68% of cells showed amplification of the *FLT3* gene and two copies of the 13q21.31 region. The remaining cells showed a normal hybridization pattern. The FISH result was nuc ish (*FLT3* amp, CON13×2) [136/200] (Figures 1(e) and 1(f)). FISH results confirmed the presence of *FLT3* amplification in this patient. The patient was treated with standard chemotherapy of 4 cycles of 5-azacytidine (50 mg/m^2^ 7 days per cycle). Follow-up cytogenetic studies were performed. BM aspirate appeared to show a decreased blast (7%) compared to the previous marrow, with no evidence of progression to acute leukemia. The karyotype result revealed 46,XY,i(17)(q10),del(20)(q11.2q13.3)[20] which is considered to be the same as the original abnormalities. FISH identified 10% cells with dmin. No dmin chromosome was identified in the metaphase. The patient now has been living for 11 months.

## 3. Discussion

Extrachromosomal dmin and intrachromosomal homogeneous staining regions (HSRs) are two types of cytogenetic hallmarks of gene amplification [10]. Gene amplification may increase the abnormal expression and activation of oncogenes in the pathogenesis of tumors [11]. Dmin is a rare cytogenetic abnormality in hematological malignancies, and most of the case reports on dmin are in patients with myeloid neoplasms [12]. Molecular investigation of dmins in myeloid neoplasms revealed that the most commonly reported dmin is due to the amplification of *MYC* gene [5]. Dmin harboring MLL gene has also been reported as well as some with amplification of other genes such as C8FW, C-ETS1, HTRX-1, and PVT-1 [5, 13, 14].

The mechanisms of dmin formation remain unclear. The episome model including chromosome excision, cyclization, recombination, and amplification has been proposed to explain the genesis [3]. Some of the studies on MYC amplification as dmin revealed the cryptic deletion of 8q24, and amplification of deleted segment was identified subsequently, which may support the above hypothesis [15]. However, some cases could not detect the deletion segments, like our case, which indicates that other mechanisms may be involved in the generation of dmin or the excision event was postreplicative [16, 17].

The FLT3 protein is encoded by a gene located on chromosome 13q12 and has a tyrosine-protein kinase activity [18]. By binding to its ligand, *FLT3* receptor phosphorizes multiple cytoplasmatic proteins and activates several downstream signaling pathways, such as the STAT-5, Ras/Raf/MAPK, and PI3 kinase cascades. All the pathways play vital roles in the promotion of cell cycle progression, inhibition of apoptosis, and activation of differentiation [19].

In the TCGA data set, *FLT3* amplification can be found in various solid tumors such as colon cancer, stomach cancer, prostate cancer, and breast cancer. A case reported by Moreira et al. [20] described a refractory metastatic colorectal cancer patient with *FLT3* amplification. The patient showed significant improvement after the target therapy of *FLT3* inhibitor, sorafenib. Lim et al. [9] reported a patient with sigmoid colon cancer and lung metastasis. *FLT3* amplification was also identified; then, regorafenib was given for 12.4 months with partial response. However, when patient-derived tumor cells from colon cancer with *FLT3* amplification were used for further study, tumor growths could not be inhibited by either regorafenib or sorafenib. A study conducted by Jiang et al. [21] identified a signaling circuit between *MYC* and *FLT3* via miR-150 in AML with *MLL* rearrangements underlying the pathogenesis of leukemia. We hypothesize that *MLL/FLT3/MYC* may have oncogene activity and may have a continuous network effect on tumorigenesis, leading to genomic instability.

Genetic aberration of *FLT3* mutation has a high frequency of onset in myeloid neoplasm. The oncogenic role of *FLT3* in myeloid neoplasms usually results from activating somatic mutations including length mutation (LM)/internal tandem duplication (ITD) in the juxtamembrane domain and point mutations within the tyrosine kinase domain (TKD) [22]. Current research results indicate that *FLT3-ITD* may lead to unfavourable impact on the prognosis especially a high relapse rate in patients with myeloid neoplasm, while the impact of *FLT3-TKD* on the prognosis is still unclear [23]. Mutant *FLT3* is often expressed at higher levels, which may lead to constitutive phosphorylation and activation of downstream signaling [24]. *FLT3* amplification could attribute to the high expression of *FLT3* gene resulting in a poor prognosis.

In our case, array CGH revealed that the amplified segment within 13q12 is a 2 Mb region that contains several OMIM genes. *FLT3*, as one of the most meaningful oncogenes in hematological malignancies, is involved in this amplification. A commercially available FISH probe of *FLT3* was used to confirm the amplified region. Our findings suggest that, besides the oncogenic activation of *FLT3-ITD/TKD* mutations observed in myeloid neoplasm, amplification involving FLT3 may be another mechanism contributing to leukemogenesis. Since the current case is supposed to be the first to describe the amplification of *FLT3* in myeloid neoplasms, it is hard to tell the further mechanism among these oncogenes. More data is needed to explore the complex internal relationship.

Due to the important role of *FLT3* in hematological disorders, applications of targeted therapies including *FLT3* inhibitors and tyrosine kinase inhibitors have been developed to inhibit the activation of *FLT3* [8, 22]. However, the insufficient efficacy, acquisition of resistance, and therapy-related toxicities make the option of treatment still elusive [19].

CMML belongs to myeloproliferative/myelodysplastic syndromes (MPN/MDS), as defined by the World Health Organization (WHO) classification of myeloid neoplasms in 2016. Up to date, allogeneic stem cell transplantation (HSCT) remains the best choice for long-term survival. Also, hypomethylating agents like azacitidine (AZA) and decitabine have been approved for the treatment [25]. There are only a few cases that described dmin in CMML [6, 26, 27]. The prognostic significance of dmin in CMML is not well understood. The reported cases have only survived for a few months. Although some patients' condition deteriorated rapidly, one patient treated with azacitidine achieved complete response despite progression of disease later [26]. In our case, using 5′-AZA showed a significant decrease in *FLT3*-dmin, indicating that the hypomethylation agents may have effect on inhibition of *FLT3* amplification. A recent study suggested that combination of 5′-AZA associated with *FLT3* inhibitor leads to a high antileukemic activity, which represents a novel approach to target *FLT3/ITD* mutated AML patients [28]. Additional clinical trials will be required to explore this field, with the hope that *FLT3* inhibitors will play a positive role along with the conventional chemotherapies for the treatment of myeloid neoplasm.

Currently, dmin is generally considered to be associated with poor clinical outcomes [16]. However, some case reports indicated that accompanied cytogenetic aberrations may affect the overall prognosis in addition to the dmin. A study conducted by Bruckert et al. [29] suggested that dmin combined with trisomy 4 or loss of a single X chromosome presented good response to chemotherapy and prolonged survival. As for the patients harboring dmin accompanied by complex chromosome aberrations, disease progression and short survival can be easily seen [5, 30]. Our case patient showed additional cytogenetic abnormalities of i(17) and del(20) along with dmin at the same time. Isolated isochromosome 17q case was predominantly related to a high rate of progression to AML in previous studies [31], while deletion of 20q does not show any survival effect on myeloproliferative disease [32]. This can also explain why the patient only gets partial remission after several cycles of treatment. In a word, additional cytogenetic abnormality besides dmin should be taken into account for predicting the prognosis.

Our case presented as CMML with *FLT3* amplification in the form of dmin. We efficiently confirm the *FLT3* amplification by performing FISH using a specific *FLT3/*CON13 probe. To the best of our knowledge, it is the first report identifying the *FLT3*-dmin in CMML. Genomic alteration of oncogenes via dmin is a rare phenomenon, which may be a target for basic therapeutic approaches, as well as a new direction for studying the pathogenesis of leukemia. It is apparent that more extensive mappings of dmin in hematologic disorders are necessary.

## Data Availability

The data that support the findings of this study are available from the corresponding author upon reasonable request.
